# Young Male Patients with Atrial Fibrillation and CHA_2_DS_2_-VASc Score of 1 May Not Need Anticoagulants: A Nationwide Population-Based Study

**DOI:** 10.1371/journal.pone.0151485

**Published:** 2016-03-17

**Authors:** Yi-Hsin Chan, Lung-Sheng Wu, Shang-Hung Chang, Hsin-Fu Lee, Jia-Rou Liu, Lai-Chu See, Yung-Hsin Yeh, Chi-Tai Kuo

**Affiliations:** 1 The Cardiovascular Department, Chang Gung Memorial Hospital, Linkou, Taoyuan, Taiwan; 2 College of Medicine, Chang Gung University, Taoyuan, Taiwan; 3 Department of Public Health, College of Medicine, Chang Gung University, Taoyuan, Taiwan; 4 Biostatistics Core Laboratory, Molecular Medicine Research Center, Chang Gung University, Taoyuan, Taiwan; 5 Division of of Rheumationalogy and Allergy and Immunology, Department of Internal Medicine, Chang Gung Memorial Hospital, Linkou, Taoyuan, Taiwan; University Hospital Medical Centre, GERMANY

## Abstract

**Background:**

It is unclear whether oral anticoagulants are beneficial for atrial fibrillation (AF) patients with low CHA_2_DS_2_-VASc score. Age could be important in determining the risk of thromboembolism in low risk AF patients (CHA_2_DS_2_-VASc score of 1 for male or 2 for female).

**Methods:**

The Taiwan National Health Insurance Research Database (NHIRD) was used and 27,521 AF patients with CHA_2_DS_2_-VASc score of 1 (male) or 2 (female) not receiving anticoagulants were acquired as the study cohort, which were classified into three age groups: 20–49, 50–64, and 65–74 years. The clinical endpoint was the occurrence of ischemic thromboembolism within one year of follow up.

**Results:**

During the follow-up of 0.94 ± 0.19 years, 385 (2.19%) male patients experienced ischemic thromboembolism, with annual rate of 2.32%. The annual risk ranged from 1.29%, 2.43% to 2.77% for male patients aged 20–49, 50–64 and 65–74 years respectively. Of the female patients, 218 (2.20%) experienced clinical event with annual rate of 2.32%. The annual risk increased from 1.87%, 2.28% to 2.64% for female patients aged 20–49, 50–64 and 65–74 years respectively. There was no difference in risk between the male patients aged 20–49 years with CHA_2_DS_2_-VASc score of 1 and overall male patients with CHA_2_DS_2_-VASc score of 0. (*P* = 0.631) The female patients aged 20–49 years with CHA_2_DS_2_-VASc score of 2 was associated with a higher risk of thromboembolic events than overall female patients with CHA_2_DS_2_-VASc score of 1 (HR = 1.93; *P* = 0.008).

**Conclusions:**

Age is important in determining the risk of thromboembolism in AF patients with single risk factor. In male patients <50 years old with CHA_2_DS_2_-VASc score of 1, the risk of ischemic thromboembolism was low. Considering the benefits and the risk of bleeding, oral anticoagulation therapy may not be favorable in these patients.

## Introduction

Atrial fibrillation (AF) is the most common cardiac arrhythmia and causes a 4- to 5-fold increased risk of thromboembolic stroke.[1] Current guidelines for oral anticoagulants (OACs) advocate using the CHA_2_DS_2_-VASc (heart failure, hypertension, age 75 years or older, diabetes mellitus, previous stroke or transient ischemic attack (TIA), vascular disease, age 65 to 74 years, female gender) scoring system to stratify the risk of stroke.[2–6] The guidelines virtually recommend anticoagulants for AF patients with a CHA_2_DS_2_-VASc score of 2 and above. However, the recommendations for AF patients with CHA_2_DS_2_-VASc score of 1 are not consistent. The ESC, Asia Pacific Heart Rhythm Society, and National Institute for Health and Care Excellence guidelines suggest OACs for AF patients with CHA_2_DS_2_-VASc score of 1 which is not due to gender (female).[7–9] On the other hand, the 2014 ACC/AHA guidelines do not firmly recommend OACs for these low risk patients (class IIb).[10] The actual risk of ischemic thromboembolism and whether or not anticoagulants should be given to these AF patients remain uncertain.

Limited data are available for AF patients with minimal risk factors at age less than 65 years old. In young people, risk factors in CHA_2_DS_2_-VASc score may not be as important as in elderly people. We hypothesized that the risk of ischemic thromboembolism in AF patients under the age of 65 is low when only one risk factor in CHA_2_DS_2_-VASc score exists. Therefore, this study was to investigate the one-year risk of ischemic thromboembolism in young AF patients with single risk factor not due to female (CHA_2_DS_2_-VASc score of 1 for male or 2 for female) and the impact of different component risk factors.

## Materials and Methods

### Study Population

The NHI system is a mandatory universal health insurance program which offers comprehensive medical care coverage to all Taiwanese residents. The NHIRD is released by the National Health Research Institutes of Taiwan, and is a national database consisting of detailed health care data from >23 million enrollees, representing >99% of the population of Taiwan. This study was exempted from review by the Chang Gung medical foundation institutional review board (104-1177B) because the original identification number of each patient in the NHIRD is encrypted and de-identified to protect their privacy, using a consistent encrypting procedure so that it was feasible to link and continuously follow all of the claims belonging to the same patient within the NHIRD.

### Study Cohort

**Fig 1:**
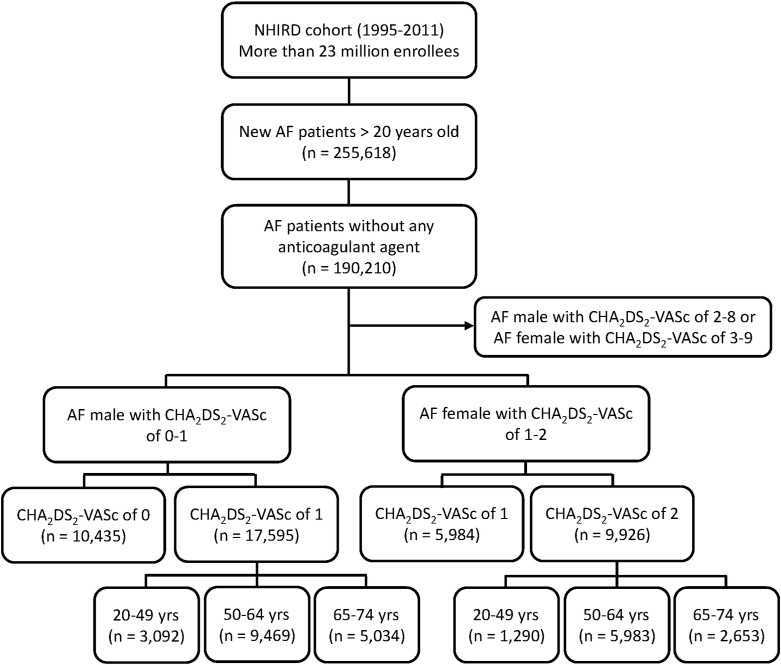
Flow Chart of Study Cohort Enrollment. Patients who received treatments with warfarin or any antiplatelet agent, including aspirin and clopidogrel, were excluded from the study population. A total of 190,210 patients were finally enrolled in the study cohort with 17,595 males with CHA_2_DS_2_-VASc score of 1 and 9,926 females with CHA_2_DS_2_-VASc score of 2. Among the 17,595 male patients, there were 3,092, 9,469, and 5,034 patients in the age groups of 20 to 49, 50 to 64, and 65 to 74 years of age, respectively. Among the 9,926 female patients, there were 1,290, 5,983, and 2,653 patients in the age groups of 20 to 49, 50 to 64, and 65 to 74 years of age, respectively. There were other 10,435 males with CHA_2_DS_2_-VASc score of 0 and 5,984 females with CHA_2_DS_2_-VASc score of 1 used as the reference group, respectively. AF = atrial fibrillation; CHA_2_DS_2_-VASc = heart failure, hypertension, age 75 years or older, diabetes mellitus, previous stroke/transient ischemic attack, vascular disease, age 65 to 74 years, female.

From January 1, 1995, to December 31, 2011, a total of 255,618 patients with AF who were older than 20 years of age were identified in the NHIRD. AF was determined using International Classification of Diseases, ninth revision, Clinical Modification (ICD-9-CM) code (427.31). To ensure diagnostic accuracy, patients were defined as having AF only when there was a discharge diagnosis or more than two outpatient visits related to AF. The diagnostic accuracy of AF based on the definition was validated before.[11, 12] The patients who received treatment with warfarin or any antiplatelet agent including aspirin and clopidogrel were excluded from the study population. A total of 190,210 AF patients were finally enrolled as the study cohort, including 17,595 males with a CHA_2_DS_2_-VASc score of 1 and 9,926 females with a CHA_2_DS_2_-VASc score of 2. Among the 17,595 male patients, there were 3,092, 9,469, and 5,034 patients in the age groups of 20 to 49, 50 to 65, and 65 to 74 years of age, respectively. Among the 9,926 female patients, there were 1,290, 5,983, and 2,653 patients in the age groups of 20 to 49, 50 to 64, and 65 to 74 years of age, respectively. Another 10,435 males had a CHA_2_DS_2_-VASc score of 0 and 5,984 females had a CHA_2_DS_2_-VASc score of 1, and they served as the reference group. We defined the young age group in the range of 20 to 49 years of age, because ischemic strokes attacked after adolescence and before the 50 years of age are typically considered as “young stroke” based on previous definition.[13] A flowchart of the enrollment of the study cohort is shown in Fig 1.

### Calculation of CHA2DS2-VASc Score and Determination of the Endpoint

The CHA_2_DS_2_-VASc score was calculated for each patient by assigning 1 point each for an age between 65 and 74 years, a history of heart failure, hypertension, diabetes, vascular disease (myocardial infarction or peripheral artery disease), and female gender, and 2 points each for a history of an ischemic stroke, TIA, or an age more than 75 years. The ICD-9-CM codes used to identify the risk factor components of the scoring scheme are shown in S1 Table. To ensure diagnostic accuracy, patients were defined as having ischemic stroke, TIA, myocardial infarction, peripheral artery disease and heart failure only when it was a discharge diagnosis. The diagnoses of hypertension and diabetes were considered when the diagnostic codes were confirmed more than twice in outpatient visits. The diagnostic accuracies of comorbidities with hypertension, diabetes mellitus, congestive heart failure, myocardial infarction, and peripheral vascular disease had been validated before.[14, 15] The clinical endpoints were defined as the occurrence of ischemic stroke or systemic embolism with concomitant imaging studies of the brain or target organs, including computed tomography, magnetic resonance imaging and ultrasound imaging. The study endpoint of ischemic stroke/systemic embolism was considered only when it was a discharge diagnosis. The accuracy of the diagnosis of ischemic stroke in the NHIRD has been validated in previous studies, with a positive predictive value of 88.4% and sensitivity of 97.3%.[15, 16]

### Statistical Analysis

Data are presented as mean values ± standard deviation for continuous variables, and proportions for continuous variables. The risk of ischemic thromboembolism was assessed using Cox regression analysis. The curve of ischemic thromboembolism-free rate after AF diagnosis was plotted using the Kaplan-Meier method, with statistical significance examined by the log-rank test. Statistical significance was defined as a *P* value of less than 0.05. All statistical analyses were performed using SAS 9.4 (SAS Institute Inc., Cary, NC, USA).

## Results

The annual risk of ischemic thromboembolism of the 190,210 study patients and the corresponding CHA_2_DS_2_-VASc scores are summarized in Table 1. As expected, the annual risk of a clinical event increased with increases in CHA_2_DS_2_-VASc score, with an annual risk of 1.18% for a CHA_2_DS_2_-VASc score of 0 to as high as 20.26% for a CHA_2_DS_2_-VASc score of 7 or higher. In order to investigate the risk of ischemic stroke/systemic emboli in AF patients by age and gender, we further separated the total AF patient group into four age subgroups in both genders: 20 to 49, 50 to 64, 65 to 74, and more than 75 years (Table 2). The results showed that were distinct annual risks of clinical events even with the same CHA_2_DS_2_-VASc score in each gender. It is noted that AF patients who were younger than 50 years of age had the lowest annual risk of clinical events compared to the older age groups in both genders with one additional risk factor (CHA_2_DS_2_-VASc score of 1 for male or 2 for female). We also found that young females with a CHA_2_DS_2_-VASc score of 6 (47.71%) carried an unusual high annual risk of clinical events, which may have been due to the limited number of patients and clinical events in these young patient groups.

**Table 1:** AnnuaI Risk of Ischemic thromboembolism by CHA_2_DS_2_-VASc Score among AF patients without anticoagulants.

	Ischemic thromboembolsim		
	Events	Person-years	Risk
CHA_2_DS_2_-VASc			
0	116	9822	1.18%
1	440	22233	1.98%
2	1114	31787	3.50%
3	2017	40572	4.97%
4	2570	36162	7.11%
5	2155	20197	10.67%
6	1425	8610	16.55%
7–9	828	4087	20.26%

AF = atrial fibrillation; CHA_2_DS_2_-VASc = heart failure, hypertension, age 75 years or older, diabetes mellitus, previous stroke/transient ischemic attack, vascular disease, age 65 to 74 years, female

**Table 2:** AnnuaI Risk of Ischemic Thromboembolism Stratified by CHA_2_DS_2_-VASc Score, Gender, and Age among AF patients without anticoagulants.

	Age = 20–49		Age = 50–64		Age = 65–74		Age = 75 up		All	
	Events	Risk	Events	Risk	Events	Risk	Events	Risk	Events	Risk
Female										
0	- -	- -	- -	- -	- -	- -	- -	- -	- -	- -
1	14	0.56%	41	1.30%	- -	- -	- -	- -	55	0.78%
2	23	1.87%	129	2.28%	66	2.64%	- -	- -	218	1.94%
3	22	5.75%	111	3.31%	327	3.60%	184	5.63%	644	3.16%
4	7	11.68%	85	11.45%	394	5.59%	885	6.14%	1371	4.19%
5	2	12.76%	62	21.90%	282	12.87%	888	7.38%	1234	5.04%
6	1	47.71%	13	20.61%	210	21.76%	775	14.59%	999	7.19%
7–9	0	0.00%	7	59.02%	65	23.17%	647	20.98%	719	9.56%
Male										
0	22	0.50%	94	1.75%	- -	- -	- -	- -	116	1.18%
1	38	1.29%	216	2.43%	131	2.77%	- -	- -	385	2.32%
2	41	4.73%	205	4.56%	446	3.68%	204	4.14%	896	4.00%
3	19	9.10%	163	12.97%	440	5.91%	751	4.82%	1373	5.61%
4	4	8.79%	90	17.93%	410	15.47%	695	6.50%	1199	8.63%
5	2	12.37%	31	34.08%	245	22.12%	643	14.43%	921	16.24%
6	0	0.00%	6	17.87%	53	21.72%	367	18.45%	426	18.78%
7–9	- -	- -	- -	- -	14	20.30%	95	14.81%	109	15.35%

AF = atrial fibrillation; CHA_2_DS_2_-VASc = heart failure, hypertension, age 75 years or older, diabetes mellitus, previous stroke/transient ischemic attack, vascular disease, age 65 to 74 years, female

Male patients with a CHA_2_DS_2_-VASc score of 0 had a relatively low annual risk of clinical events with 1.18% (Table 2). Of note, the annual risk of clinical events remained low (1.29%) in male patients with a CHA_2_DS_2_-VASc score of 1 who were less than 50 years of age. The older AF male patients (more than 50 years) with a CHA_2_DS_2_-VASc score of 1 and overall male patients with a CHA_2_DS_2_-VASc score of more than 1 had a significantly increased annual risk of clinical events (more than 2.43%). In contrast to the male patients, the young female patients with a CHA_2_DS_2_-VASc score of 2 had an increased annual risk of clinical events (1.87%) as compared with the overall female patient groups with a CHA_2_DS_2_-VASc score of 1 (0.97%).

We then specifically focused on the patients with a CHA_2_DS_2_-VASc score of1 for males or 2 for females, since the recommendations for anticoagulation treatment in patients with a low CHA2DS2-VASc score remain unclear based on the current guidelines. Table 3 summarizes the baseline data of the patient groups with one single additional stroke risk factor. The proportions of each age group (20 to 49, 50 to 64 and 65 to 74 years) to all AF patients were 17.6%, 53.8%, and 28.6% in the males with a CHA_2_DS_2_-VASc score of 1, and 13.0%, 60.2%, and 26.7% in the females with a CHA_2_DS_2_-VASc score of 2, respectively. The mean age and prevalence of each risk factor in the study patients by age group are shown in Table 3**.** The mean ages were 42.9 ± 5.8, 58.0 ± 4.2, and 69.7 ± 2.8 for the male patients and 43.6 ± 5.5, 58.3 ± 4.2, and 69.5 ± 2.8 for the female patients in the 20 to 49, 50 to 64 and 65 to 74 years age groups, respectively. Hypertension and vascular diseases were the most and least prevalent risk factors for the AF patients aged 20 to 49 and 50 to 64 years in both genders, respectively. Of note, the young AF patients had a higher prevalence of congestive heart failure compared with the older AF patients with the same CHA_2_DS_2_-VASc score in both genders (12.3% vs. 6.3% for the males; 15.0% vs. 5.0% for the females).

**Table 3:** Baseline Characteristics for AF Patients with One Additional Risk Factor of CHA_2_DS_2_-VASc Score*.

		Male			Female	
Onset age of AF	20–49	50–64	65–74	20–49	50–64	65–74
Number	3,092 (17.6%)	9,469 (53.8%)	5,034 (28.6%)	1,290 (13.0%)	5,983 (60.3%)	2,653 (26.7%)
Age, years	42.9 ± 5.8	58.0 ± 4.2	69.7 ± 2.8	43.6 ± 5.5	58.3 ± 4.2	69.5 ± 2.8
CHA2DS2-VASc score	1	1	1	2	2	2
Risk factors according to CHA2DS2-VASc:						
Heart failure	381(12.3%)	601 (6.3%)	0 (0%)	193 (15.0%)	301 (5.0%)	0 (0.0%)
Hypertension	2351(76.0%)	7738 (81.7%)	0 (0%)	898 (69.9%)	5052 (84.4%)	0 (0.0%)
Age 65–74 years	0 (0.0%)	0 (0.0%)	5034 (100%)	0 (0.0%)	0 (0.0%)	2653 (100%)
Diabetes mellitus	316 (10.2%)	965 (10.2%)	0 (0.0%)	190 (14.7%)	606 (10.1%)	0 (0.0%)
Vascular disease	44 (1.4%)	165 (1.7%)	0 (0.0%)	9 (0.7%)	24 (0.4%)	0 (0.0%)

*CHA_2_DS_2_-VASc score of 1 for males and 2 for females

AF = atrial fibrillation; CHA_2_DS_2_-VASc = heart failure, hypertension, age 75 years or older, diabetes mellitus, previous stroke/transient ischemic attack, vascular disease, age 65 to 74 years, female

**Fig 2:**
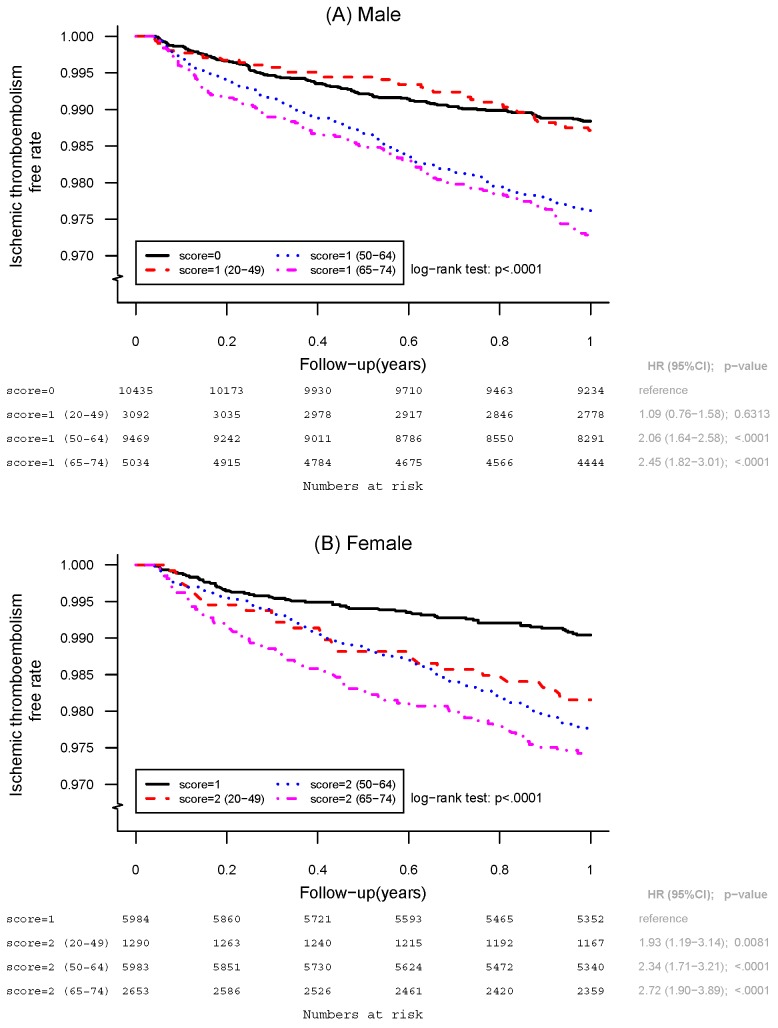
Kaplan-Meier Survival Curves in AF patients with One Additional Risk Factor of CHA_2_DS_2_-VASc Score. **A.** Cumulative survival curves in male patients with CHA_2_DS_2_-VASc Score of 1 in the age groups of 20 to 49, 50 to 64, and 65 to 74 years of age, respectively. There is no difference of the cumulative risk of ischemic thromboembolism between male AF patients with CHA_2_DS_2_-VASc score of 1 for 20 to 49 years of age and overall patients with CHA_2_DS_2_-VASc score of 0. **B.** Cumulative survival curves in female patients with CHA_2_DS_2_-VASc Score of 2 in the age groups of 20 to 49, 50 to 64, and 65 to 74 years of age, respectively. Young female patient with CHA_2_DS_2_-VASc score of 2 and 20 to 49 years of age showed significantly higher cumulative risk of clincial event as compared with overall female patients with CHA_2_DS_2_-VASc score of 1.

Fig 2 shows the ischemic thromboembolism-free curves of the AF patients with one additional risk factor for three age subgroups. Using the patients overall without any additional risk factors (CHA_2_DS_2_-VASc score of 0 for males or 1 for females) as the reference group in each gender, the male patients with a CHA_2_DS_2_-VASc score of 1 in both the 50 to 64 and 65 to 74 years age groups had a significantly higher cumulative risk of ischemic stroke/systemic embolism compared with the reference group (HR, 2.06; 95% CI, 1.64–2.58; *P* < 0.0001 for 50 to 64 years of age; HR, 2.45; 95% CI, 1.82–3.01; *P* < 0.0001 for 65 to 74 years of age). However, the young male patients with a CHA_2_DS_2_-VASc score of 1 did not have a higher cumulative risk of clinical events than the male patients overall with a CHA_2_DS_2_-VASc score of 0 (HR, 1.09; 95% CI, 0.76–1.58; *P* = 0.6313). In addition, the 50 to 64 and 65 to 74 years old patients had a significantly higher risk compared with the 20 to 49 years old patients with a CHA_2_DS_2_-VASc score of 1 (HR, 1.88; 95% CI, 1.33–2.65; *P* = 0.0003 for 50 to 64 versus 20 to 49 years of age; HR, 2.14; 95% CI, 1.49–3.07; *P* < 0.0001 for 65 to 74 versus 20 to 49 years of age).

For female gender, the female patients with a CHA_2_DS_2_-VASc score of 2 for all age subgroups had a significantly higher risk compared with the female patients overall with a CHA_2_DS_2_-VASc score of 1 (HR, 1.93; 95% CI, 1.19–3.14; *P* = 0.0081 for 20 to 49 years of age; HR, 2.34; 95% CI, 1.71–3.21; *P* < 0.0001 for 50 to 64 years of age; HR, 2.72; 95% CI, 1.90–3.89; *P* < 0.0001 for 65 to 74 years of age) compared with the reference group. Of note, neither the 50 to 64 nor 65 to 74 years old patients had a significantly higher risk compared with the 20–49 years old patients with a CHA_2_DS_2_-VASc score of 2 (HR, 1.21; 95% CI, 0.78–1.89; *P* = 0.3916 for 50 to 64 versus 20 to 49 years of age; HR, 1.41; 95% CI, 0.88–2.26; *P* = 0.1575 for 65 to 74 versus 20 to 49 years of age).

Among the 3,092 male patients with a CHA_2_DS_2_-VASc score of 1 who were younger than 50 years of age, only heart failure (HR, 3.34; 95% CI, 1.92–5.82; *P* < 0.0001) had a significantly higher risk of thromboembolic events compared with the reference group (Table 4). For the 1,290 female patients with a CHA_2_DS_2_-VASc score of 2 who were younger than 50 years of age, both heart failure (HR, 2.88; 95% CI, 1.15–7.20; *P* = 0.0235) and hypertension (HR, 1.92; 95% CI, 1.10–3.35; *P* = 0.0216) have a significantly higher risk of a clinical event compared with the reference group. Among the 9,469 male patients with a CHA_2_DS_2_-VASc score of 1 who were 50 to 64 years of age, all four risk factors (heart failure, hypertension, diabetes, and vascular disease) contributed to a significant risk of a clinical event compared with the reference group (all *P* < 0.01). For the 5,983 female patients with a CHA_2_DS_2_-VASc score = 2 who were 50 to 64 years of age, heart failure, diabetes and hypertension all had a significant risk of a clinical event compared with the reference group (all *P* < 0.05).

**Table 4:** Risk of Ischemic Thromboembolism in AF Patients with One Additional Risk Factor of CHA_2_DS_2_-VASc Score*.

Age	Risk Factor Components of the CHA_2_DS_2_-VASc Score	Patients	Events	Person-Years	Annual Risk (%) (95% CI)	Hazard Ratio (95% CI)	*P* Value
**CHA**_**2**_**DS**_**2**_**-VASc Score of 1 for Males**							
20–49	Heart failure	381	14	354.79	3.95% (1.88%-6.01%)	3.34 (1.92–5.82)	<0.0001
	Diabetes mellitus	316	2	297.28	0.67% (0.08%-2.43%)	0.57 (0.14–2.30)	0.4298
	Hypertension	2351	22	2249.83	0.98% (0.57%-1.39%)	0.83 (0.53–1.31)	0.4210
	Vascular disease	44	0	40.37	0.00% (- -)	- -	- -
50–64	Heart failure	601	19	552.94	3.44% (1.89%-4.98%)	2.89 (1.78–4.70)	<0.0001
	Diabetes mellitus	965	20	898.07	2.23% (1.25%-3.20%)	1.88 (1.17–3.02)	0.0092
	Hypertension	7738	170	7289.03	2.33% (1.98%-2.68%)	1.97 (1.56–2.50)	<0.0001
	Vascular disease	165	7	156.07	4.49% (1.80%-9.24%)	3.81 (1.78–8.17)	0.0006
65–74	—	5034	131	4735.07	2.77% (2.29%-3.24%)	2.34 (1.82–3.01)	<0.0001
**CHA**_**2**_**DS**_**2**_**-VASc Score of 2 for Females**							
20–49	Heart failure	193	5	177.64	2.81% (0.91%-6.57%)	2.88 (1.15–7.20)	0.0235
	Diabetes mellitus	190	2	182.02	1.10% (0.13%-3.97%)	1.13 (0.28–4.64)	0.8628
	Hypertension	898	16	859.12	1.86% (0.95%-2.77%)	1.92 (1.10–3.35)	0.0216
	Vascular disease	9	0	8.08	0.00% (- -)	- -	- -
50–64	Heart failure	301	15	277.73	5.40% (2.67%-8.13%)	5.54 (3.13–9.81)	<0.0001
	Diabetes mellitus	606	12	577.08	2.08% (0.90%-3.26%)	2.14 (1.15–4.00)	0.0168
	Hypertension	5052	102	4789.16	2.13% (1.72%-2.54%)	2.19 (1.58–3.04)	<0.0001
	Vascular disease	24	0	23.67	0.00% (- -)	- -	- -
65–74	—	2653	66	2499.41	2.64% (2.00%-3.28%)	2.72 (1.90–3.89)	<0.0001

*CHA_2_DS_2_-VASc score of 1 for males and 2 for females

AF = atrial fibrillation; CHA_2_DS_2_-VASc = heart failure, hypertension, age 75 years or older, diabetes mellitus, previous stroke/transient ischemic attack, vascular disease, age 65 to 74 years, female

## Discussion

This study is a nation-wide large population-based investigation to analyze the risk of ischemic thromboembolism focusing on CHA_2_DS_2_-VASc score of 1 in males and 2 in females, specifically in patients < 65 years old with AF who did not receive OACs. We found that age is an important factor in determining the risk of stroke in these low risk patients who were younger than 65 years old. The risk of ischemic thromboembolism in male patients < 50 years old was extremely low (less than 1.29%) for those with a CHA_2_DS_2_-VASc score of 1. The cumulative event rate was similar between the young male patients with a CHA_2_DS_2_-VASc score of 1 and the male patients overall with a CHA_2_DS_2_-VASc score of 0 (*P* = 0.6313). In contrast, the young female patients with a CHA_2_DS_2_-VASc score of 2 had an increased risk of clinical events compared with the female patients overall with a CHA_2_DS_2_-VASc score of 1 (*P* = 0.0081). Since the risk of major bleeding due to non-vitamin K antagonist OACs has been reported to be as high as 0.25% to 1.45% per year and even higher for warfarin,[17–21] the actual benefit of anticoagulation therapy is questionable in young male AF patients with one risk factor in CHA_2_DS_2_-VASc score. For male patients with 50 to 64 years of age and female patients with all age groups carrying one single additional stroke risk factor, we observed that OACs is still recommended, since the annual risk of ischemic thromboembolism was more than 1.87% and the cumulative event rate remained significantly high in these AF patients compared to the reference group (Fig 2). Heart failure rather than other risk factors including diabetes, hypertension and vascular disease was the most important risk factor for thromboembolism in the young AF patients with one additional risk factor of the CHA_2_DS_2_-VASc score in both genders. Since the recommendations for anticoagulation treatment in patients with a CHA_2_DS_2_-VASc score of 1 are not consistent between the current guidelines, our observations should help to improve the rationale of using OACs in patients < 50 years old or between 50–65 years old with CHA_2_DS_2_-VASc score of 1 not due to gender.

Age has been reported to be a more important non-modifiable risk factor for ischemic stroke compared to other factors.[22] Several possible mechanisms may explain the vulnerability to ischemic stroke due to aging. Aging is often associated with other independent risk factors such as hypertension, diabetes, vascular disease, or heart failure in the CHA_2_DS_2_-VASc scoring system, and the CHA_2_DS_2_-VASc score only reflects the presence or absence of risk factors rather than the actual impact caused by the duration of the risk factor itself. The same risk factor in older AF patients may therefore have a more obvious impact on the risk of stroke than in younger AF patients, since the same risk factor may have already existed in the older AF patients for more than several decades. Old age is associated with the severity of vascular endothelial dysfunction and contributes to the formation of vascular atherosclerosis and abnormal homeostasis.[23–29] Therefore, aging itself may have a more important impact in contributing to the risk of ischemic stroke and disease severity compared to other risk factors. Our results indicated a cut-off point in the cumulative event rate of ischemic stroke/systemic emboli between the male AF patients younger than 50 years of age and those 50 to 64 years of age (Fig 2). Considering the risk of major bleeding increases with the duration of anticoagulation therapy, it is essential to incorporate a young age into the scoring scheme to assess the rationale of very long-term anticoagulation therapy for young AF patients compared to older AF patients.

Not all risk factors in the CHA_2_DS_2_-VASc score were associated with an equal risk in the AF patients. For the AF patients with single risk factor not due to female (CHA_2_DS_2_-VASc score of 1 for males and 2 for females), the risk factor of an age of 65 to 74 years carried a significant risk of a clinical event in both genders (HR, 2.34; 95% CI, 1.82–3.01; *P* < 0.0001 for males; HR, 2.72; 95% CI, 1.90–3.89; *P* < 0.0001 for females). For the 9,469 male patients with a CHA_2_DS_2_-VASc score of 1 who were 50 to 64 years of age, the HRs of the four risk factors (congestive heart failure, diabetes, hypertension and vascular disease) were comparable (Table 4). For the 5,983 female patients with a CHA_2_DS_2_-VASc score of 1 who were 50 to 64 years of age, heart failure had the highest risk of a clinical event (HR, 5.54; 95% CI, 3.13–9.81; *P* < 0.0001), while vascular disease did not contribute to a higher risk of a clinical event in these patients. Our findings showed that for AF patients with vascular disease alone, the risk of ischemic thromboembolism was significantly increased by 3.81-fold for the male patients aged 50 to 64 years, while it did not increase the risk of clinical events for male patients aged 20 to 49 years or for female patients aged 20 to 49 and 50 to 64 years. The number of young patients with vascular disease was thus limited in our study cohort, which may have decreased the statistical power of vascular disease in predicting the risk of ischemic thromboembolism.

Hypertension and diabetes have been independently and consistently associated with ischemic stroke in AF patients in previous studies.[30–32] Although the presence of congestive heart failure would be expected to increase the risk of ischemic stroke in AF patients based on current pathophysiological concepts, previous studies have indicated that a history of congestive heart failure does not seem to be an independent risk factor for thromboembolism in AF patients.[30–32] However, those studies mainly focused on AF patients overall, and the findings may not be the same when focusing on young AF patients. Our results revealed that congestive heart failure rather than other risk factors such as diabetes, hypertension and vascular disease was the most important risk factor for thromboembolism in young AF patients with one additional risk factor of ischemic stroke in both genders. In addition to the presence or absence of hypertension or diabetes as recorded in the CHA2DS2-VASc score, the severity of hypertension and the duration of diabetes may also influence the absolute stroke rate in AF patients with these disorders. The severity and duration of hypertension or diabetes in young AF patients would be expected to be much lower than in older AF patients, which may explain why these risk factors did not play an important role in determining the risk of thromboembolic events in the young AF patients compared with the older patients. Since the recommendations for anticoagulation treatment in patients with a low CHA2DS2-VASc score are unclear based on the current guidelines, the presence of congestive heart failure may help to assess the rationale of using oral anticoagulants in young AF patients with one additional risk factor.

### Limitations

Intrinsic limitations exist in nation-wide cohort study like this. This is registry data and therefore the diagnosis is not strictly re-examined. In addition, the CHA_2_DS_2_-VASc score was fixed once the patient had been diagnosed with AF in this study. However, the CHA_2_DS_2_-VASc score may change dynamically with time.

## Conclusions

Age is an important factor in determining the risk of ischemic thromboembolism in AF patients with low CHA_2_DS_2_-VASc scores (1 for male or 2 for female). The annual and cumulative risks of ischemic thromboembolism in male AF patients less than 50 years old were very low and similar to the AF patients without any additional risk factors (CHA_2_DS_2_-VASc score = 0). Considering the benefits and costs including the risk of bleeding, oral anticoagulation therapy may not be favorable or should be used individually in these patients.

## Supporting Information

S1 Table International Classification of Disease, 9th edition, Clinical Modification (ICD 9-CM) Codes Used to Define Risk Factors of CHA_2_DS_2_-VASc Score and Clinical Outcome in the Study Cohort. (DOC)
