# Supplementary material for: Young Male Patients with Atrial Fibrillation and CHA2DS2-VASc Score of 1 May Not Need Anticoagulants: A Nationwide Population-Based Study
Source: PLoS One. 2016 Mar 17;11(3):e0151485. doi: 10.1371/journal.pone.0151485 (PMC4795759; doi:10.1371/journal.pone.0151485)
Supplement: S1 Table — (DOC) [file pone.0151485.s001.doc]

**S1 Table. International Classification of Disease, 9^th^ edition, Clinical Modification (ICD 9-CM) Codes Used to Define Risk Factors of CHA_2_DS_2_-VASc Score and Clinical Outcome in the Study Cohort.**

| Disease | ICD-9 Codes | Diagnosis definition |
| --- | --- | --- |
| Atrial fibrillation (AF) | 427.31 | Discharge or outpatient department ≥2 |
| Ischemic stroke | 433, 434, | Discharge |
| Systemic embolism | 444 | Discharge |
| Hemorrhagic stroke | 430, 431, 432 | Discharge |
| [Transient ischemic attack](http://en.wikipedia.org/wiki/Transient_ischemic_attack) | 435 | Discharge |
| Peripheral arterial occlusive disease | 440.2 | Discharge |
| Myocardial infarction | 410, 412 | Discharge |
| Congestive heart failure | 428 | Discharge |
| Hypertension | 401, 402 | Outpatient department ≥2 |
| Diabetes mellitus | 250 | Outpatient department ≥2 |
|  |  |  |

AF = atrial fibrillation; CHA_2_DS_2_-VASc = heart failure, hypertension, age 75 years or older, diabetes mellitus, previous stroke/transient ischemic attack, vascular disease, age 65 to 74 years, female
